# Monitoring of sirolimus in the whole blood samples from pediatric patients with lymphatic anomalies

**DOI:** 10.1515/med-2023-0652

**Published:** 2023-03-02

**Authors:** Natalia Treder, Alina Plenis, Olga Maliszewska, Natalia Kaczmarczyk, Ilona Olędzka, Piotr Kowalski, Tomasz Bączek, Ewa Bień, Małgorzata Anna Krawczyk, Anna Roszkowska

**Affiliations:** Department of Analytical Chemistry, Medical University of Gdansk, Gdansk, Poland; Department of Pharmaceutical Chemistry, Medical University of Gdansk, Gdansk, Poland; Department of Pediatrics, Hematology and Oncology, Medical University Gdansk, Gdansk, Poland

**Keywords:** SIR, whole blood samples, DLLME, LC-MS/MS, therapeutic drug monitoring

## Abstract

In recent years, *off-label* use of sirolimus (SIR) has been gaining attention in the clinical practice. However, since it is critical to achieve and maintain therapeutic blood levels of SIR during treatment, the regular monitoring of this drug in individual patients must be implemented, especially in *off-label* indications of this drug. In this article, a fast, simple, and reliable analytical method for determining SIR levels in whole blood samples is proposed. Sample preparation based on dispersive liquid–liquid microextraction (DLLME) followed by liquid chromatography-mass spectrometry (LC-MS/MS) was fully optimized toward the analysis of SIR and proposed as a fast, simple, and reliable analytical method for determining the pharmacokinetic profile of SIR in whole-blood samples. In addition, the practical applicability of the proposed DLLME-LC-MS/MS method was evaluated by analyzing the pharmacokinetic profile of SIR in whole blood samples obtained from two pediatric patients suffering from lymphatic anomalies, receiving this drug as *off-label* clinical indication. The proposed methodology can be successfully applied in routine clinical practice for the fast and precise assessment of SIR levels in biological samples, thus allowing SIR dosages to be adjusted in real time during pharmacotherapy. Moreover, the measured SIR levels in the patients indicate the need for monitoring between doses to ensure the optimal pharmacotherapy of patients.

## Graphical abstract

**Figure:**
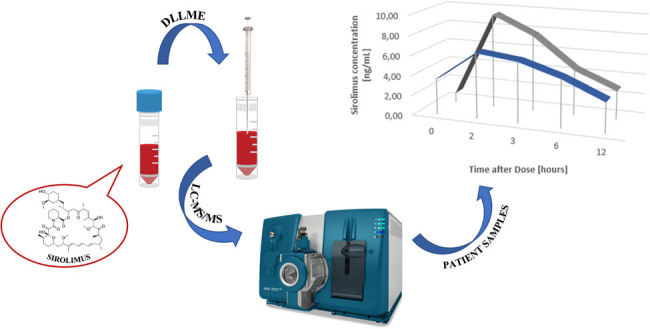

## 1 Introduction

Sirolimus (SIR) (also known as rapamycin) is one of the most widely used immunosuppressive drugs. One reason for its widespread use is its ability to bind and form complexes with the FKBP12 protein, which allows it to suppress the PI3K-Akt-mammalian target of rapamycin (mTOR) signaling pathway, thus inhibiting cell growth, proliferation, and differentiation [1]. In addition, since SIR targets mTOR in mammals, it also affects the expression of the vascular endothelial growth factor responsible for angiogenesis and lymphangiogenesis [2]. Due to these inhibitory effects, the use of SIR in the prophylaxis of organ rejection in renal transplant patients aged 13 years or older has been approved by the Food and Drug Administration (FDA) since 1999 [3], and its use in the polytherapy of lymphangioleiomyomatosis in pediatric patients has been approved since 2003 [4]. Furthermore, SIR’s antiproliferative effects have led to its use for *off-label* applications, including the treatment of various types of cancers [5,6] and vascular anomalies [7,8,9]. For instance, a prospective screening of patients with generalized lymphatic anomaly (GLA) and Gorham–Stout disease revealed that incorporating SIR in therapeutic treatment can help to stabilize or reduce the symptoms of the disease [7]. Phase II trials have also confirmed the efficacy and safety of SIR in patients with lymphatic malformation (LM) and venous malformation resistant to standard therapy [10]. Nevertheless, it should be emphasized that such uses of SIR are new and that available dosing guidelines are based on the existing experimental protocols; thus, it is especially critical to monitor and adjust therapeutic regimens of this drug during *off-label* applications.

Pharmacokinetic studies of SIR, which are largely based on its administration in renal transplantation, have revealed that it has low oral bioavailability (∼15%), with even lower bioavailability among black patients [11,12]. Due to SIR’s narrow therapeutic index and high interindividual and intraindividual pharmacokinetic differences among patients, it is important to maintain its concentration within the recommended therapeutic range of 5–15 ng/mL (based on data from transplant patients), as concentrations outside of this range may result in acute transplant rejection (<5 ng/mL) or serious side effects, such as thrombocytopenia, leukopenia, or hypertriglyceridemia (>15 ng/L) [13,14]. In addition, SIR is also a substrate for cytochrome P450 3A4 enzymatic complexes in the liver; therefore, many drugs that act as inhibitors/inducers of CYP3A4 may interact with SIR and enhance its side effects [15]. Moreover, it may be even more difficult to predict the pharmacokinetics of SIR in *off-label* applications for treating disorders in children (e.g., lymphatic anomalies), as they possess different LADME processes related to the pharmacokinetics of drugs, including SIR, than adults [16].

The literature contains only a few studies related to the therapeutic monitoring of SIR concentrations in real blood samples [12,17,18,19,20]. These studies utilize both enzyme immunoassays and chromatographic methods [12,17,18,19] and capillary electrophoresis (CE) [20] to measure the levels of SIR in biofluids. While the quickness and simplicity of enzyme immunoassays are distinct advantages, many researchers have observed cross-reactions between antibodies and SIR metabolites when using this method, which creates the risk of overestimating the SIR concentration. In addition, studies comparing enzyme-linked immunoassays and chromatographic/mass spectrometry have found that SIR levels measured with immunoassay tests were higher when both methods were used to analyze the same samples. Zhao et al. reported over 60% overestimation of the results obtained from the enzyme multiplied immunoassay technique (EMIT) in comparison to the liquid chromatography-mass spectrometry (LC-MS/MS) method during SIR determinations [21]. The issues related to the correlation of results between chromatographic methods and immunoassay tests in the determination of immunosuppressants were also revealed by Polledri et al. In the described study, the results from LC-MS/MS analysis were compared with the results from immunoassay tests, and the overestimation of the results in the case of the latter one was confirmed [22]. On the other hand, while chromatographic techniques allow for much greater sensitivity and selectivity for target compounds, it remains a challenge to develop fast and low-cost procedures using this technology. Moreover, the isolation and the analysis of SIR in therapeutic drug monitoring (TDM) is challenging and complicated, as SIR binds strongly to red blood cells (RBCs) (∼95%), which creates the need for a hemolysis step in whole blood testing. Thus, although significant advances in mass spectrometry have enabled sensitive and reproducible results, there is still a need to develop new sample preparation approaches that will allow the efficient hemolysis of RBCs and the isolation of SIR from whole blood samples, in addition to simple and reproducible performance between laboratories. Such novel approaches would help to overcome the problem of false-positive results frequently reported for immunological tests, as well as providing researchers and clinicians with a simple and precise methodology for monitoring real concentrations of SIR in biological matrices. With the exception of one study that utilized CE with an ultraviolet (UV) detector [20], the previous work in this analytical area has exclusively employed LC/MS, which confirms the universality of this technique in current laboratory practice [23,24,25,26,27]. Obviously, the use of LC-MS/MS may be more challenging than immunoassay tests in typical laboratories, in terms of both operation and maintenance costs. However, the use of TDM is recommended for a wide range of pharmaceuticals, which makes LC-MS/MS an appealing option, as it is able to monitor a range of drugs in patients simultaneously, including SIR [28].

The aim of this study is to develop a simple, fast, and sensitive analytical method based on LC-MS/MS that can be applied for the TDM of SIR in whole blood samples. The developed method was validated, and its usefulness for SIR monitoring was confirmed by applying it to analyze the pharmacokinetic profile of SIR in real whole-blood samples acquired from two pediatric patients with rare diseases (lymphatic anomalies) treated with SIR in off-label applications. The resultant SIR concentration profiles were then determined and compared with those of other pharmacokinetic studies of SIR. To the best of our knowledge, the proposed method is the first to combine dispersive liquid–liquid microextraction (DLLME) sample preparation and LC-MS/MS analysis for the profiling of SIR levels in the whole blood. Furthermore, this study is also the first to monitor the SIR pharmacokinetic profiles of pediatric patients with lymphatic anomalies at hourly intervals.

## 2 Materials and methods

### 2.1 Chemicals and reagents

EVE, which was used as an internal standard (IS), and SIR were obtained from Sigma-Aldrich (St. Louis, MO, USA), while LC-MS-grade methanol (MeOH), acetonitrile (ACN), and formic acid (FA) were supplied by Merck (Darmstadt, Germany). Ethanol (EtOH) and chloroform (Chl) were purchased from POCH (Gliwice, Poland) and Chempur (Poland), respectively. The ammonium acetate (AA) used to prepare the 200 mM ammonium formate stock solution was obtained from Sigma-Aldrich (St. Louis, MO, USA), and all water used in the experiments was purified with a milli-Q system (Molsheim, France). The 1,000 and 250 μL syringes (Hamilton) were supplied by Sigma-Aldrich (St. Louis, MO, USA), and the control whole blood samples were obtained from healthy volunteers.

### 2.2 Standard solutions

A stock solution of SIR and IS was prepared in EtOH at a concentration of 1 mg/mL, and working standard solutions of SIR at concentration levels of 0.1, 0.5, 1, 5, and 10 µg/mL were prepared by dissolving an appropriate volume of the stock solution in the working solutions. A working standard solution of IS at a concentration of 10 µg/mL was prepared by dissolving an appropriate volume of IS stock solution in EtOH. Both the stock and working SIR and IS solutions were stored at −80°C until use.

### 2.3 Preparation of calibration and quality control (QC) samples

Whole blood calibration samples with SIR concentrations in the range of 1–50 ng/mL and IS at 50 ng/mL were prepared via hemolysis, followed by DLLME to ensure the recovery of any erythrocyte-bound immunosuppressant. The optimization of the DLLME procedure has been described in detail in our previous article [29]. Briefly, 250 µL of whole blood was spiked with appropriate volumes of SIR calibration standards at concentrations of 0.1, 0.5, 1, 5, and 10 µg/mL and IS calibration standards at 10 µg/mL to obtain final concentrations of 1, 2.5, 5, 10, 25, and 50 ng/mL for SIR and 50 ng/mL for IS, respectively. Next, 750 µL of water was added to the enriched samples, with the resultant mixture being subjected to sonication for 20 s. The samples were then centrifuged (10 min, 4,000 rpm), and the obtained supernatant was transferred to Eppendorf tubes. After the hemolysis step, the DLLME procedure was performed. In this process, 1 mL of the extraction mixture, which consisted of 800 µL of EtOH and 200 µL of Chl, was rapidly injected into 1 mL of supernatant using a microsyringe (1,000 µL Hamilton) to create a cloudy solution. The samples were then stored at −80°C for 3 min to enhance the formation of droplets, followed by centrifugation at 3,000 rpm for 5 min. The droplets at the bottom of the tube (150 ± 20 µL) were collected and transferred to a different Eppendorf tube using a microsyringe (250 µL Hamilton), where they were evaporated to dryness at 45°C under vacuum conditions using a CentriVap (Labconco, Kansas City, Missouri, USA). The dry residue was reconstituted in 50 µL of a water/MeOH mixture (50:50, v/v) containing 5 mM AA, and the resultant solution was transferred to the inserts and analyzed via LC-MS/MS.

Whole blood QC samples at concentrations of 1.00 (lower limit of quantification [LLOQ]), 2.50 (low limit of quantification [LQC]), 10.00 (middle limit of quantification [MQC]), or 25 ng/mL (high limit of quantification [HQC]) also were prepared using the described procedure earlier.

### 2.4 Instrumentation and chromatographic conditions

All analyses were conducted on a Nexera HPLC system equipped with a CT-20AC column oven, an LC-30AD solvent delivery unit, and an SIL-30AC thermostatic autosampler. Chromatographic separation was achieved on a YMC-Triart C8 column (50 × 2.1 mm, 5 µm) (YMC, Kyoto, Japan) maintained at a temperature of 45°C. The mobile phases consisted of MeOH (phase A) and water (phase B) with the addition of 0.1% FA and 10 mM AA solution. The gradient elution had a flow rate of 0.55 mL/min and utilized the following program: 0–0.20 min 80% A; 0.20–1.20 min 100% A; 1.20–1.31 min 80% A; and 1.31–2.50 min 80% A (re-equilibration). The injection volume was set at 1 µL, and the autosampler temperature was held at 4°C.

The chromatographic instrument was coupled to a mass spectrometer Sciex QTrap 6500 (AB Sciex, Concorde, Ontario, Canada) equipped with an electrospray ionization interface operated in a positive ionization mode, with all analyses being performed in a multiple reaction monitoring mode. The ion spray voltage was set at 5,500 V, and the temperature was set at 300°C. The curtain gas, ion source gas 1, and gas 2 were set at 20, 50, and 40 psi, respectively, and dwell time was set at 150 ms for each transition. The settings for SIR and the IS are presented in Table 1. Finally, the integrated data system used in this work was the MultiQuant 3.0.1. software package.

**Table 1:** The mass spectrometry conditions for the analysis of SIR and IS

Parameters	SIR	IS
Precursor ion (Da)	931.527	975.537
Product ion (Da)	864.300	908.400
DP (V)	26.000	36.000
CE (V)	25.000	25.000
CXP (V)	20.000	52.000

DP – declustering potential; CE – collision energy; CXP – collision exit potential.

### 2.5 Assay validation

The proposed analytical method for determining SIR in whole blood was validated according to the guidelines for bioanalytical methods [30,31]. To this end, the following parameters were evaluated: linearity, limits of detection (LOD), limits of quantification (LOQ), selectivity, accuracy, precision, stability, carry-over effects, and recovery. The validation parameters were determined using calibration and QC samples of whole blood.

#### 2.5.1 Linearity

Linearity was determined by analyzing six calibration series of whole blood samples (each in triplicate) within a concentration range of 1–50 ng/mL. All series were made and measured within 1 day. The obtained results were used to calculate a calibration curve based on a linear regression model (*y* = *bx* + *a*), where *y* is the ratio of the SIR to IS areas versus SIR concentration of SIR (*x*) in ng/mL, *b* is the slope of the line, and *a* is the *y* intercept.

#### 2.5.2 LOD and LOQ

The LOD and the LOQ were calculated according to the following equations:

(1) {\rm{LOD}}=3.3\frac{{Sa}}{b},]

(2) {\rm{LOQ}}=10\frac{{Sa}}{b},]

where *Sa* is the standard deviation (SD) of the intercept and *b* is the slope of the calibration curve.

#### 2.5.3 Selectivity

Selectivity was estimated by analyzing a blank whole blood sample (i.e., unspiked) and samples that had been spiked with SIR at 25 ng/mL and IS at 50 ng/mL. In accordance with FDA and International Conference on Harmonization (ICH) guidelines, we assessed whether there was any interference in the analyte and IS retention times based on the matrix peaks.

#### 2.5.4 Accuracy and precision

Accuracy and precision were measured by analyzing QC samples enriched with SIR at four concentration levels – namely, 1 (LLOQ), 2.5 (LQC), 10 (MQC), and 25 ng/mL (HQC) – and IS at a constant concentration of 50 ng/mL. All analyses were performed in triplicate. Measurements of the QC samples were performed on the same and different days of the experiments to estimate intra- and inter-day accuracy and precision. Accuracy was defined as the percentage recovery of the analyte in relation to the nominal concentration of the analyte spiked into the sample, while precision was expressed by the relative standard deviation (RSD (%)). The obtained data were then compared with FDA and ICH criteria.

#### 2.5.5 Stability

The stability of SIR in whole blood was tested by analyzing low, middle, and high QCs containing the same SIR concentrations used to determine the precision and accuracy of the method (i.e., 2.5, 10, and 25 ng/mL). Before analysis, the spiked samples were stored under various thermal conditions – namely, 4 h at room temperature (∼25°C), 2 months at −80°C, and 24 h at 4°C – to test their short-term, long-term, and postpreparative stability (autosampler conditions). In addition, the QC samples were stored at −80°C and allowed to thaw to room temperature in a triple cycle to determine the effect of freezing and thawing on SIR stability. The results obtained for these samples were compared with the results for fresh QC samples, and the back-calculated SIR concentration levels were then estimated. Based on the criteria for bioanalytical methods, the samples were determined to be stable, as the SIR concentration at each level was within 100 ± 15% of the nominal concentration, while precision expressed by the RSD (%) value was ≤15%.

#### 2.5.6 Carry-over

A carry-over test was performed to detect the presence of any residual analyte in the apparatus after the sample had been analyzed. For this purpose, the analysis of samples enriched in SIR and IS at a concentration of 50 ng/mL was performed before the analysis of the blank sample (i.e., without the presence of SIR and IS).

#### 2.5.7 Recovery

The absolute recovery of the analytes from whole blood samples for the developed DLLME-based analytical method was calculated after the analysis of spiked samples to which calibration standards were added before and after extraction (2.5 and 25 ng/mL for SIR and 50 ng/mL for the IS). Once again, each sample was measured in triplicate. Thus, the obtained ratio of peak area responses in the samples spiked pre-extraction to those spiked postextraction enabled the evaluation of the absolute recovery results for the target analytes.

### 2.6 Collection of patient whole blood samples

Whole blood samples were collected from two pediatric patients in the Department of Pediatrics, Hematology and Oncology, at the University Clinical Center in Gdansk. The study was approved by the Ethics Committee of the Medical University of Gdansk, and the donors’ parents signed a consent form, agreeing to their child’s participation in the study. The conditions of the patients were established through medical history, as well as clinical and laboratory examinations.

Patient 1 was a 3-year-old boy with a severe form of a GLA with left chylothorax. *Off-label* SIR therapy was initiated bi-daily with an oral dose of 0.8 mg/m^2^ and gradually increased until a therapeutic blood concentration of 10–15 ng/mL had been reached. This required more than 2 months of treatment. The whole blood samples used to establish the SIR concentration profile in this study were collected from this patient once they had progressed to taking an SIR oral suspension at a dose of 1.9 mg every 12 h, which was approximately 3 months after the initiation of treatment. The whole blood samples were collected over 2 days at the following time intervals: (1) before the morning dose; (2) 2 h after the morning dose; (3) 3 h after the morning dose; (4) 6 h after the morning dose; (5) 12 h after the morning dose and immediately before the evening dose; (6) 24 h after the first and immediately before the second morning dose; (7) 26 h after the first and 2 h after the second morning dose; and (8) 27 h after the first dose and 3 h after the second morning dose. In addition to SIR, the patient’s therapy included the intake of zoledronic acid at a dose of 0.05 mg/kg every 4 weeks along with a nonsteroidal anti-inflammatory drug (metamizole sodium) and a calcium supplement, as well as 4,000 IU of cholecalciferol orally once per day. The SIR level for this patient before the administered drug dose was determined to be 14.2 ng/mL using an immunochemical test.

Patient 2 was an 11-year-old girl with generalized lymphadenopathy, immune thrombocytopenia, and autoimmune anemia (genetic tests indicated a very rare type 3 dyserythropoietic anemia). SIR was included in the patient’s treatment in the same manner as it would be used for an autoimmune lymphoproliferative syndrome. To achieve the recommended therapeutic concentration of 5–10 ng/mL, the initial SIR dose was set at 1 mg/m^2^ orally once per day and gradually increased to 2 mg/m^2^ once per day over a 2-month period. The samples that were used to establish the SIR concentration profile were collected when the patient was taking the target dose of the immunosuppressant. As with Patient 1, seven samples were collected from Patient 2 within 2 days: (1 A) before the first dose; (2 A) 2 h after the first dose; (3 A) 3 h after the first dose; (4 A) 6 h after the first dose; (5 A) 12 h after the first dose; (6 A) 24 h after the first dose and immediately before the second dose; and (7 A) 26 h after the first dose and 2 h after the second dose. Apart from SIR, Patient 2 was also receiving 2,000 IU of cholecalciferol orally once per day. The SIR concentration for this patient before the administered drug dose was determined to be 5.7 ng/mL using an immunochemical test.

## 3 Results

### 3.1 Method validation

**Figure 1:**
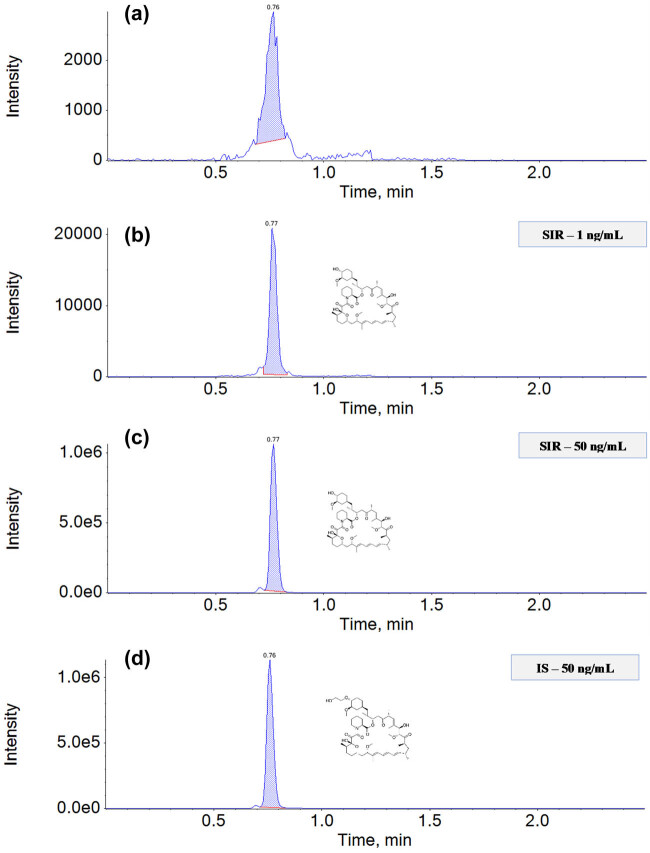
LC-MS/MS selected ion chromatogram with [M + NH_4_]^+^-signals of (a) blank whole blood; (b) whole blood sample spiked with SIR at a concentration of 1 ng/mL; (c) whole blood sample spiked with SIR at a level of 50 ng/mL; and (d) whole blood spiked with IS at a level of 50 ng/mL.

The analysis of the calibration samples confirmed the developed method’s linearity over the entire concentration range of 1–50 ng/mL (six series of measurements performed in triplicate). The calibration curve equation was *y* = 0.019 (±0.0010)*x* + 0.0064 (±0.00004), where *y* and *x* were the peak area ratio of SIR/IS and the SIR concentration, respectively. In addition, the prepared calibration curve had a correlation coefficient (*R* ^*2*^) of ≥0.999. The LOD and LOQ were calculated using the calibration curve equation and were found to be 0.2 and 0.6 ng/mL, respectively. Chromatograms were obtained after the analysis of blank whole blood samples and after the analysis of blood samples spiked with SIR and enriched with ISs at concentrations of 25 ng/mL (SIR) and 50 ng/mL (IS); these chromatograms showed no interference from SIR vs IS and SIR/IS vs background signals (Figure 1), which confirmed the proposed method’s selectivity. The analysis of the LLOQ and three QC samples (LQC, MQC, and HQC) showed that the intraday and interday precision for the LLOQ were 5.48 and 8.80, respectively, while the intraday and interday accuracy were 95.67 and 93.02%, respectively (Table 2). In the case of the QC samples, the intraday and interday precision values ranged from 3.11 to 5.73% and 0.23 to 5.28%, respectively. The intraday and interday accuracy were less than 99.52 and 99.32%, respectively. These results confirm that the precision and accuracy of the developed analytical method are consistent with the applicable standards of bioanalytical methods. The validation also included a stability test of the low, middle, and high QCs (2.5, 10, 25 ng/mL). The obtained results for the storage of SIR and the IS at various thermal conditions (as detailed in Section 2.5.5) clearly show that SIR in whole blood remains stable under all temperature conditions, including after freezing and thawing cycles (Table S2). The obtained RSD (%) was ≤9.21%, while the accuracy was from 94.48 to 108.40%, which indicates that the results of the SIR and IS concentration determinations do not depend on the conditions under which the samples are stored before analysis. These results are consistent with previous reports [32,33]. In addition, the carry-over effect was examined to determine whether there was analyte transfer between analyses due to the presence of residues in the elements of the apparatus. The analytical signals obtained for a blank sample analyzed after the sample with the highest SIR concentration (50 ng/mL) were evaluated at 1.95 ± 0.02% of the peak area obtained after the analysis of the sample with the lowest analyte concentration (1 ng/mL); thus, carry-over effects did not affect the accuracy of the measurements or the measured SIR concentrations. The mean absolute recoveries for SIR and the IS from whole blood samples were 65.57 ± 5.24% and 67.27 ± 4.66%, respectively.

**Table 2:** Intraday and interday precision and accuracy for the quantification of SIR in whole blood samples by the LC-MS/MS method (*n* = 6)

Intraday (*n* = 6)					Interday (*n* = 6)			
QCs	Concentration (ng/mL)		Precision RSD (%)	Accuracy (%)	Concentration (ng/mL)		Precision RSD (%)	Accuracy (%)
	Spiked (ng/mL)	Found (mean ± SD)			Spiked (ng/mL)	Found (mean ± SD)		
LLOQ	1	0.96 ± 0.05	5.48	95.67	1	0.93 ± 0.08	8.80	93.02
LQC	2.5	2.59 ± 0.15	5.73	103.64	2.5	2.55 ± 0.13	5.28	101.93
MQC	10	10.08 ± 0.47	4.65	100.84	10	9.93 ± 0.15	1.56	99.32
HQC	25	24.88 ± 0.77	3.11	99.52	25	25.01 ± 0.06	0.23	100.04

#### 3.1.1 Real sample monitoring

**Figure 2:**
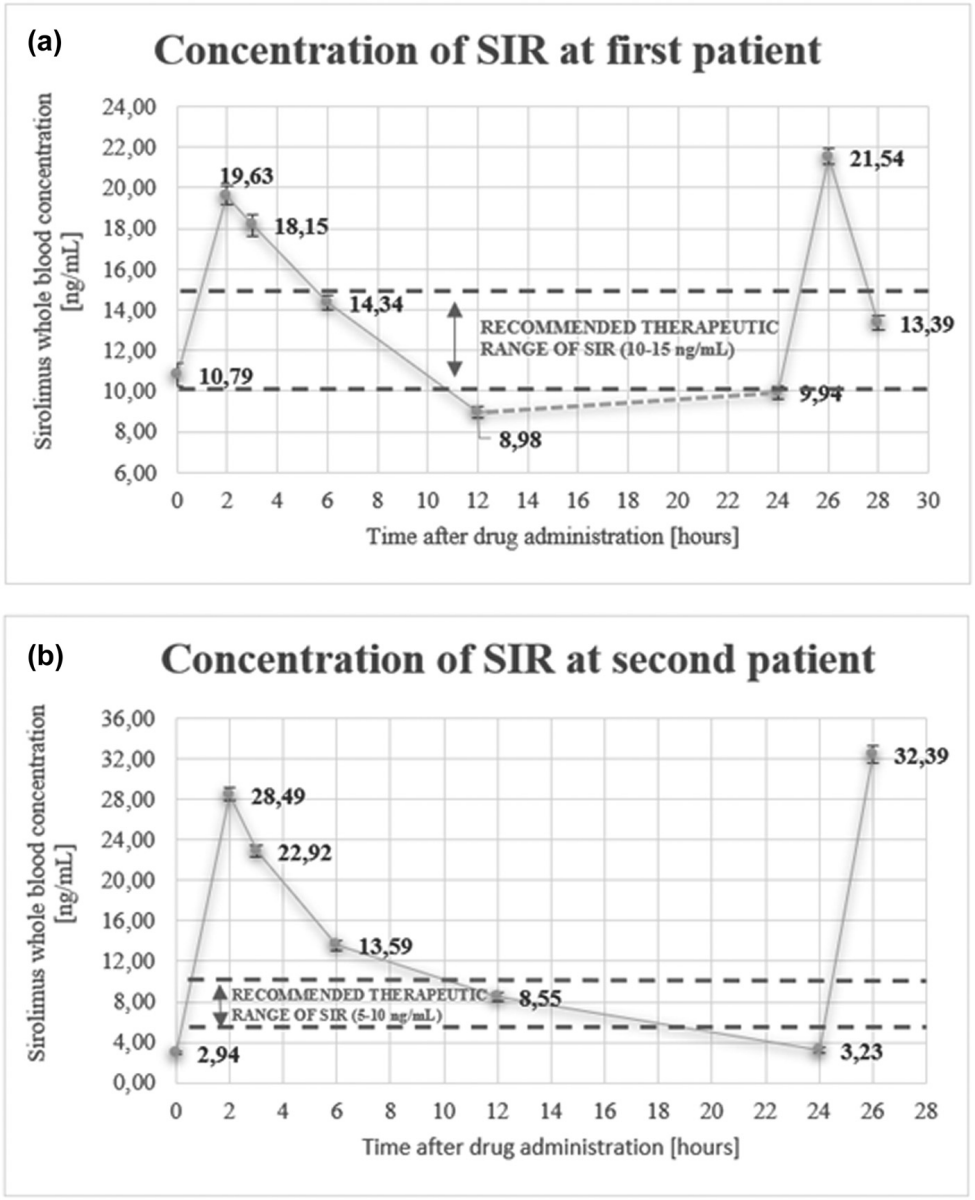
Sirolimus concentration in whole blood versus time profile for (a) Patient 1 taking 1.9 mg of SIR once every 12 h and (b) Patient 2 taking 2.0 mg of SIR once every 24 h. The dashed line indicates the time period during which no samples were taken.

The developed and validated DLLME-LC-MS/MS method was used to determine the SIR concentration profile in samples from two oncologic pediatric patients (Section 2.6). In the case of Patient 1, who was taking 1.9 mg dose of SIR orally every 12 h, the peak concentrations found in the samples collected 2 h after drug intake were as follows: 19.63 ± 0.48 ng/mL after the morning dose on the first day and 21.54 ± 0.41 ng/mL after the morning dose on the second day (Figure 2a). In the hours following the collection of the first sample on the morning of Day 1, the SIR concentration in the whole blood samples decreased consistently (3 h—18.15 ± 0.54 ng/mL; 6 h—14.34 ± 0.37 ng/mL), reaching the trough concentration (8.98 ± 0.29 ng/mL) 12 h after administration. The trough concentration of SIR 12 h after the evening dose (i.e., the next dose) was equal to 9.94 ± 0.32 ng/mL.

For Patient 2, who was taking a 2.0 mg dose of SIR orally once every 24 h, the overall concentration profile of the drug was slightly different. Similar to Patient 1, the highest measured SIR concentration in whole blood samples from Patient 2 was observed 2 h after intake. However, due to the different dose levels and regimens, Patient 2’s SIR levels were 28.49 ± 0.66 ng/mL 2 h after intake on the first day, and 32.39 ± 0.86 ng/mL on the second day of sampling (Figure 2b). Further differences in the SIR pharmacokinetic profiles of the two patients were attributable to the different dosing regimens (i.e., Patient 1 took SIR twice per day, while Patient 2 only received SIR once per day). For Patient 1, the lowest SIR concentration was found in the samples collected 12 h after intake, while the SIR concentration in Patient 2 was found to decrease during the 24 h after intake. The rate of decrease in Patient 2 was as follows: 22.92 ± 0.54 ng/mL at 3 h; 13.59 ± 0.49 ng/mL at 6 h; 8.55 ± 0.41 ng/mL at 12 h; and 3.23 ± 0.23 ng/mL at 24 h after administration.

## 4 Discussion

In current clinical practice, enzyme-linked immunoassays are preferred over mass spectrometry-based methods for the fast analysis of SIR levels in biological samples; however, enzyme-linked immunoassays suffer from many limitations, particularly by the cross reactions with SIR metabolites and other potential matrix components. Such cross reactions may affect the obtained results, for example, by overestimating data concerning SIR concentrations in real biological samples. Therefore, more precise alternative methods, such as mass spectrometry techniques, should be considered as promising options for overcoming the limitations of typical protocols for analyzing the pharmacokinetics of SIR.

In this study, an analytical method comprising sample preparation via DLLME followed by LC-MS/MS analysis was presented as a novel approach for the isolation and the quantification of SIR in complex matrices. This microextraction procedure for the isolation of SIR from whole blood samples was applied for the first time as previous reports concerning the sample preparation step were mostly based on conventional approaches, which suffers from several drawbacks (Table S1). On the contrary, DLLME is widely recognized as an eco-friendly modification of traditional LLE that is characterized by its simplicity and significantly reduced consumption of organic solvents. In the case of the developed DLLME procedure, the consumption of organic solvent was low when the time required for sample preparation per 1 sample including both hemolysis step and DLLME was only about 3 min. The literature contains only one study in which DLLME is applied for the isolation of SIR from biological fluids [34]. However, instead of using whole blood samples to determine SIR levels, the authors of this study used serum samples in developing an analytical method for the TDM of this strongly erythrocyte-bound drug. Conversely, in the current study, SIR extraction was performed using whole blood samples according to a previously developed procedure that combines DLLME with erythrocyte lysis; detailed information about the optimization steps for this procedure can be found in our previous work [29]. The method development process also included the selection of optimal chromatographic conditions to obtain the best results. To this end, we tested three different chromatographic columns: a YMC-Triart C8 column (50 mm × 2.1 mm, 5 µm) from YMC (Kyoto, Japan); a Discovery HS C18 (50 mm × 4.6 mm, 3 µm) column from Supelco (Bellefonte, USA); and a Kinetex^®^ C18 reverse-phase column (50 mm × 2.1 mm, 1.7 μm) from Phenomenex (CA, USA). The results clearly indicated that the best resolution, time retention, and signal intensity for SIR were obtained on the C8 stationary phase; thus, this column was selected for use in subsequent analyses. Moreover, to ensure the best possible conditions for the quantification of SIR, different mobile phase compositions were tested. The analysis was performed using ACN or MeOH as the organic phase in a gradient elution with different gradient settings. Ultimately, the use of MeOH as an organic phase component in the gradient mode (described in detail in Section 2.4) enabled the best chromatographic conditions such as peak shape and symmetry, short analysis times, and improved signal intensity for both SIR and the IS. In this study, EVE was used as IS, which was in line with therapeutic regimens in which both EVE and SIR are used in combination with cyclosporin A or tacrolimus, but never used simultaneously. The application of the selected conditions facilitated the determination of SIR in whole blood samples in a total analysis time of 2.5 min, which is advantage over most of the reported LC(UPLC)-MS [17,18,21,23,24,25,26,27,33] and CE-based studies [20] on SIR quantification (Table S1). In addition, the chromatographic parameters enabled low LOD and LOQ values, which were equal to 0.2 and 0.6 ng/mL, respectively. Thus, the LOQ was lower [17,20,24,25,32] or comparable [18,21,26,27,33] with previously reported protocols for the analysis of SIR in biological matrices (Table S1). What’s more, to the best of our knowledge, this is the first study to document a validation protocol for a DLLME-LC-MS/MS method for SIR analysis that was performed and calculated according to ICH and FDA requirements. Furthermore, the results of the sensitivity, selectivity, precision, accuracy, stability, recovery, and carry-over tests (detailed in Section 3) proved that the proposed method complies with the current ICH and FDA criteria for bioanalytical methods, and is therefore suitable for routine use in controlling SIR concentrations in biological samples.

The developed analytical protocol was subsequently applied for the determination of SIR levels in real blood samples obtained from two pediatric patients with lymphatic anomalies (Section 2.6). In both clinical cases, namely, GLA (Patient 1) and generalized lymphadenopathy (Patient 2), mutations in the PI3K/Akt/mTOR pathway were observed; hence, the administration of SIR (mTOR inhibitor) to treat such conditions is justified, which has led to increased interest in its use as a part of polytherapy for these disorders [7,35]. Most prior data for this orphan use of SIR have come from clinical trials focused on demonstrating its dose–response toxicity and efficacy [7,8,10]. However, high pharmacokinetic variability between patients makes it challenging to determine the correct dosage of SIR. Thus, methods enabling the precise analysis of SIR levels in blood samples from individual patients are an essential component of optimal therapeutic regimens. The monitoring of SIR concentrations is usually performed every week at the beginning of therapy, then every 2 weeks, and finally, every month once the target blood concentration of SIR has been achieved. In typical clinical protocol, a sample is taken for an immunoassay test once per day before the administration of the next dose of the drug [36,37]. Thus, in clinical practice, the dosage of SIR is adjusted to the target therapeutic range based on its levels in the blood at a single analytical time point. Unfortunately, such analysis does not provide precise information about the drug’s LADME (ADME) process, including its absorption and distribution, which may significantly affect the dosage size and frequency used in the selected dosing schedule. In the current study, the obtained results revealed that both patients reached the maximum SIR concentration 2 h after intake, with SIR absorption occurring at the same time, despite their difference in age (3 and 11 years) (Figure 2). It should also be emphasized that some differences are apparent when comparing the SIR concentrations in the samples collected for Patient 1 at the same time points on subsequent days (19.63 ± 0.48 vs 21.54 ± 0.41 ng/mL for peak concentration). During the second day of sampling, the maximum concentration (i.e., 2 h after drug intake) was higher than at the same time point on the first day; conversely, the concentration of SIR was lower 3 h after administration on the second day compared to the same time point on the first day (13.39 ± 0.36 ng/mL vs 18.15 ± 0.54 ng/mL) (Figure 2). These results may suggest that the interday profiles of SIR concentrations in the test patient may be different. In turn, significant differences were evident in the elimination stage of this drug. Specifically, while the SIR concentration profile for Patient 1 (intake every 12 h) was relatively stable, significant fluctuations in SIR levels were observed in Patient 2 (intake every 24 h). In addition, it is also interesting that, in the case of Patient 2, a similar dependence was observed in the SIR levels determined in samples taken at the same time points on different days. However, the maximum SIR concentration on the second day was higher than the maximum SIR concentration on the first day at 28.49 ± 0.66 ng/mL and 32.39 ± 0.86 ng/mL, respectively. The results obtained for both patients indicate that the concentration of SIR tends to increase on the days following administration, but decreased to very similar values after taking each dose (Figure 2). For instance, SIR levels of 2.94 ± 0.14 ng/mL and 3.23 ± 0.23 ng/mL were recorded in the second patient 24 h after administration. In addition, a *t*_1/2_ of < 12 h was achieved with the bi-daily SIR dosage regimen, and a *t*_1/2_ of < 6 h was attained with the daily dosage regimen. Thus, a bi-daily regimen appears to be the optimal dosage scheme for maintaining a steady SIR concentration during treatment. Nonetheless, it should be emphasized that several factors may have influenced the differences observed in the pharmacokinetic profiles of SIR in the two patients in this study. For instance, the ages of patients (i.e., 3 and 11 years old) and the co-administration of other drugs may have affected the obtained pharmacokinetic values and profile for SIR. As previously reported, the *t*_1/2_ of SIR in pediatric patients is shorter than in adults [16]. Indeed, the *t*_1/2_ for the 3-year-old patient taking SIR twice per day was longer than the *t*_1/2_ for the older pediatric patient taking SIR once per day. In addition, the obtained results also indicated that the SIR levels determined for both patients were frequently outside the target therapeutic window established for this drug (Figure 2). In the case of Patient 1 (dose of 1.9 mg every 12 h), the concentration value, as clinically indicated, should be within 10–15 ng/mL. However, the peak (19.63 ± 0.48 ng/mL) and trough concentrations (8.98 ± 0.29 ng/mL) measured for this patient were outside the recommended values. The same results were observed for Patient 2 (dose of 2.0 mg every 24 h), for whom the therapeutic range was set at 5–10 ng/mL. For this patient, maximal and minimal concentrations of 28.49 ± 0.66 and 2.94 ± 0.14 ng/mL were observed, respectively. Thus, the obtained results strongly indicate the presence of large fluctuations in SIR concentration at a steady state in the individual patients, and that TDM should be personalized during SIR therapy. Given that SIR concentrations outside of the recommended range can result in adverse effects, the measured concentration levels in both patients indicate the need for monitoring between doses to ensure the optimal dosage size is being administered. In addition, it should also be noted that the predose SIR concentrations determined in this study via chromatography differed from those determined in the same patients via immunochemical techniques (Section 2.6). Based on the immunochemical test, the level of SIR measured before the administration of the next dose of this drug to the first and second patient was equal to 14.2 and 5.7 ng/mL, respectively. In our MS-based methodology, SIR concentration in the samples collected from each patient at the same time point was equal to 10.79 ± 0.57 and 2.94 ± 0.14 ng/mL, respectively. Those results are in line with previous reports, suggesting the overestimation of the results when using immunoassay tests [21,22]. Specifically, the predose concentrations of SIR detected via DLLME-LC-MS/MS analysis were lower than those indicated by the immunochemical tests. In this study, about 30–50% higher levels of SIR were detected when using immunoassay test in comparison to SIR concentration found using the DLLME-LC-MS/MS method. This discrepancy in the measured concentrations of SIR was observed especially in the samples with lower levels of this drug. Thus, the risk of overestimating the SIR concentration by immunoassay tests increased with a decrease in SIR concentration. These results align with previous observations regarding the cross-reactivity of immunochemical tests, which result in overestimated SIR levels. For example, in the case of immunoassay tests, as it was mentioned earlier, over 60% overestimation of the results obtained from the EMIT in comparison to the LC-MS/MS method during SIR determinations was reported [21]. In the study described by Polledri et al., when the results from a certified *in vitro* diagnostic kit coupled to a medical device and LC-MS/MS analysis were compared with the results from immunoassay tests, the overestimation of the results in the case of immunoassay test was calculated at the level of 30% [22]. However, more comparative studies of SIR analysis via both techniques are needed to confirm the aforementioned speculations. Furthermore, this study had a number of limitations, including a small sample size (i.e., two patients); a relatively short-time monitoring time for the SIR pharmacokinetic profile; and the presence of other, nonanalytical factors mainly related to the status of the patients and their response to drug therapy (e.g., vomiting), which could also affect measured SIR levels. Therefore, it will be necessary to conduct long-term studies wherein SIR levels are determined in larger groups of patients to determine whether the findings of the present study can be applied in routine clinical practice, specifically for monitoring SIR levels in patients with lymphatic anomalies. Nevertheless, the data from previous studies do not present the SIR profile between subsequent doses as the samples were collected every few days or even months [36,37,38]. Thus, in the aforementioned studies, the level of SIR was monitored in whole blood samples collected from patients solely before the administration of the next dose of this drug, and no further analyses of the SIR profile in subsequent hours after its administration were reported.

## 5 Conclusion

This study has presented a DLLME-LC-MS/MS method for the precise determination of the pharmacokinetic profile of SIR in whole blood samples acquired from pediatric patients. The proposed DLLME protocol offers a simple and fast sample pretreatment method that can be applied in typical analytical laboratories. In addition, the use of a mass-spectrometry-based method (LC-MS/MS) to determine SIR is consistent with current practices in developing analytical methods for TDM studies. Furthermore, the development of a quick and simple analytical method that utilizes mass spectrometry also provides a promising alternative to enzyme-linked immunosorbent assays, which remain the most common method for measuring SIR levels despite their tendency to overestimate results. The validation of the proposed DLLME-LC-MS/MS protocol complies with the current requirements for bioanalytical methods; thus, the developed method can be directly applied for the therapeutic monitoring of even trace levels of SIR in clinical samples.

The practical applicability of the presented method was also demonstrated by using it to determine SIR concentration profiles in real blood samples collected at hourly intervals from two pediatric patients with lymphatic anomalies. While the determination of SIR was performed to demonstrate the proposed method’s usefulness in analyzing real biological samples, the obtained results may also be of clinical interest, as this is the first study to monitor SIR levels in an *off-label* clinical indication. To the best of our knowledge, previous studies of such orphan clinical indications of SIR in lymphatic anomalies have only monitored levels of this drug after a single dose; thus, these studies did not allow changes in the concentration of SIR between doses to be tracked during pharmacotherapy. Nevertheless, more in-depth studies of SIR’s ADME process are needed, as the present study only analyzed whole blood samples from two patients collected over a 27-hour period and did not consider other factors that might affect SIR metabolism. Therefore, more research should be conducted using the proposed DLLME-LC-MS/MS method – particularly using large samples – to gain a more robust understanding of SIR’s pharmacokinetics and to prove the developed method’s usefulness in routine clinical practice during the TDM of SIR in individual patients.

## Supplementary Material

Supplementary material
