# Supplementary material for: Monitoring of sirolimus in the whole blood samples from pediatric patients with lymphatic anomalies
Source: Open Med (Wars). 2023 Mar 2;18(1):20230652. doi: 10.1515/med-2023-0652 (PMC9982740; doi:10.1515/med-2023-0652)
Supplement: Supplementary material [file med-2023-0652-sm.pdf]

Supplementary material

Table S1: The overview of selected papers concerning the sample preparation along with analytical methods applied in the quantification of SIR in biological matrices

Matrix	Matrix volume [μL]	Extraction procedure	Analytical method	Linearity [ng/mL]	LOQ [ng/mL]	Extraction efficiency [%]	Analysis time [min]	References
Whole blood	100	SPE	LC–MS/MS	1.5–40.0	1.5	92.3	3.5	[1]
Whole blood	100	LLE	UPLC–MS/MS	—	0.5	84.07–97.95	3.0	[2]
Whole blood	1000	SPE	CE–UV	2.5–50.0	2.3	89	>25	[3]
Whole blood	100	PPt	LC–MS/MS	0.5–50.0	0.5	>80.7	3.0	[4]
Dried blood spot (DBS)	50	Hemolysis (only)	LC–MS/MS	1.2–40.0	ND	ND	3.5	[5]
Dried blood spot (DBS)	50	PPt	LC–MS/MS	1.0-50.0	1.0	>85.7	4.6	[6]
Whole blood	50	PPt	UHPLC–MS/MS	1.1–47.0	1.3	92.59	4.5	[7]
Whole blood	200	Bio-SPME	MOI–MS/MS	1.0–50.0	0.7	ND	4.5	[8]
Whole blood	50	PPt	LC–MS/MS	0.5–100.0	0.5	60.8	3.5	[9]
Whole blood	50	PPt	UPLC–MS/MS	1.52–49.50	1.58	89.9	2.5	[10]
Whole blood; Lung tissue	200	PPt	LC–MS/MS	0.5–50; 0.5–50	0.5; 0.5	>56.80; >80.00	8.5	[11]
Urine; Serum	1000; 600	DLLME	SALDI–MS	0.10–10.00 μM 0.05–2.00 μM	29 nM; 14nM	ND	ND	[12]
Whole blood	250	DLLME	LC–MS/MS	1.0–50.0	0.6	65.57	2.5	This study

PPt – protein precipitation.

ND – not detectable.

Table S2: Stability of SIR in whole blood samples under various conditions (mean ± SD, *n* = 3)

Storage conditions	QC	Conc. added (ng/mL)	Found* (ng/mL)	Precision RSD (%)	Accuracy (%)
Short-term stability (25°C, 4 h)	LQC	2.5	2.42 ± 0.15	6.20	105.68
	MQC	10	10.34 ± 0.72	6.96	103.40
	HQC	25	23.62 ± 1.05	4.44	94.48
Long-term stability (−80°C, 2 months) stability	LQC	2.5	2.71 ± 0.18	6.64	108.40
	MQC	10	9.45 ± 0.87	9.21	94.50
	HQC	25	26.16 ± 1.33	5.08	104.64
Post-preparative storage (4°C, 24 h)	LQC	2.5	2.52 ± 0.11	4.36	100.80
	MQC	10	10.80 ± 0.43	3.98	108.00
	HQC	25	24.42 ± 0.78	3.19	97.68
Three freeze-thaw cycles stability	LQC	2.5	2.66 ± 0.22	8.27	106.40
	MQC	10	9.61 ± 0.69	7.18	96.10
	HQC	25	25.56 ± 1.72	6.73	102.24

* mean ± SD value from three samples.

References

[1] Sallustio BC, Noll BD, Morris RG. Comparison of blood sirolimus, tacrolimus and everolimus concentrations measured by LC-MS/MS, HPLC-UV and immunoassay methods. Clin Biochem. 2011;231–6. doi: 10.1016/j.clinbiochem.2010.10.005.

[2] Tszyrsznic W, Borowiec A, Pawlowska E, Jazwiec R, Zochowska D, Bartlomiejczyk I, et al. Two rapid ultra performance liquid chromatography/tandem mass spectrometry (UPLC/MS/MS) methods with common sample pretreatment for therapeutic drug monitoring of immunosuppressants compared to immunoassay. J Chromatogr B. 2013;928:9–15. doi: 10.1016/j.jchromb.2013.03.014.

[3] Buchberger W, Ferdig M, Sommer R, Vo TDT. Trace analysis of rapamycin in human blood by micellar electrokinetic chromatography. Anal Bioanal Chem. 2004;380:68–71. doi: 10.1007/s00216-004-2687-x.

[4] Zhao Y, Dai H, Li Y, Zhang Y, Guo H, Ding X, et al. Comparison of LC-MS/MS and EMIT methods for the precise determination of blood sirolimus in children with vascular anomalies. Front Pharmacol. 2022;13:925018. doi: 10.3389/fphar.2022.925018.

[5] Sadilkova K, Busby B, Dickerson JA, Rutledge JC, Jack RM. Clinical validation and implementation of a multiplexed immunosuppressant assay in dried blood spots by LC–MS/MS. Clin Chim Acta. 2013;421:152–6. doi: 10.1016/j.cca.2013.02.009.

[6] Deprez S, Stove CP. Fully automated dried blood spot extraction coupled to liquid chromatography-tandem mass spectrometry for Therapeutic Drug Monitoring of Immunosuppressants. J Chromatogr A. 2021;1653:462430. doi: 10.1016/j.chroma.2021.462430.

[7] Antunes NJ, Kipper K, Couchman L, Duncan MA, Holt DW, De Nucci G, et al. Simultaneous quantification of cyclosporin, tacrolimus, sirolimus and everolimus in whole blood by UHPLC–MS/MS for therapeutic drug monitoring. Biomed Chromatogr. 2021;35:e5071. doi: 10.1002/bmc.5071.

[8] Nazdrajić E, Tascon M, Rickert DA, Gómez-Ríos GA, Kulasingam V, Pawliszyn J. Rapid determination of tacrolimus and sirolimus in whole human blood by direct coupling of solid-phase microextraction to mass spectrometry via microfluidic open interface. Anal Chim Acta. 2021;1144:53–60. doi: 10.1016/j.aca.2020.11.056.

[9] Krnáč D, Reiffová K, Rolinski B. A new HPLC-MS/MS method for simultaneous determination of Cyclosporine A, Tacrolimus, Sirolimus and Everolimus for routine therapeutic drug monitoring. J Chromatogr B. 2019;1128:121772. doi: 10.1016/j.jchromb.2019.121772.

[10] Rigo-Bonnin R, Arbiol-Roca A, Aledo-Castillo JMG, Alía P. Simultaneous measurement of cyclosporine A, everolimus, sirolimus and tacrolimus concentrations in human blood by UPLC–MS/MS. Chromatographia. 2015;78:1459–74. doi: 10.1100%2F2012%2F571201.

[11] Nguyen TTL, Duong VA, Vo DK, Jo J, Maeng HJ. Development and validation of a bioanalytical LC-MS/MS method for simultaneous determination of sirolimus in porcine whole blood and lung tissue and pharmacokinetic application with coronary stents. Molecules. 2021;26:425. doi: 10.3390/molecules26020425.

[12] Chen PS, Cheng YH, Lin SY, Chang SY. Determination of immunosuppressive drugs in human urine and serum by surface-assisted laser desorption/ionization mass spectrometry with dispersive liquid-liquid microextraction. Anal Bioanal Chem. 2015;408:629–37. doi: 10.1007/s00216-015-9145-9.
